# Case of Strangulated Femoral Hernia in the Male, Requiring Operation

**Published:** 1827-11

**Authors:** 

**Affiliations:** St. Thomas's Hospital.


*Case of Strangulated Femoral Hernia in the Male, requiring Operation, successfully treated by* Mr. Tyrrell, St. Thomas's Hospital.

July 29th, 1827.—Edmund Tapner, ætat. forty-seven, a sawyer, of florid complexion and robust habit, states that, on the morning of the 28th, he felt something give way in the right groin, while in the act of coughing violently, and, on applying his hand, for the first time perceived a swelling in that situation. During three weeks prior to this time he had laboured under diarrhœa, by which his strength had been much reduced. In the course of the day the abdomen became painful, and in the ensuing night nausea and vomiting came on, and have since continued.

On examination, a tumor, about the size of an egg, was found below Poupart's ligament in the right groin: it was somewhat moveable, tense, and extremely tender; there was some pain in the abdomen, particularly in the hypogastrium and right iliac region; on the left side was an oblique inguinal hernia, which had existed for several years, without producing any particular inconvenience; countenance anxious; pulse quick, small, and feeble; tongue white and moist; frequent vomiting and hiccough. The bowels have not been moved since the 27th. He was placed in the warm bath, and the taxis employed, without producing any alteration in the tumor.

In the absence of Mr. Travers, the patient was seen by Mr. Tyrrell early on the evening of the 29th, and, after attempting ineffectually to reduce the hernia, proposed the operation, to which the patient readily assented; and it was then performed in the usual manner. On opening the sac, a small quantity of turbid serum escaped, and a knuckle of small intestine, of a dark purple colour, was exposed, on one spot of which effusion had taken place beneath the peritoneal coat. Some slight adhesions of the intestine to the sac were easily separated; and, after dilating the stricture in a direction upwards, the bowel was readily replaced, and the wound dressed with sutures and adhesive straps. Relief was experienced very shortly, and the pulse became more full.

℞. Magnes. Sulph. ʒij.; Tr. Opii m. vj.; Aq. Menth. ℥j. M. ft. haustus statim sumendus, et secundâ quâque horâ repetend.

July 30th.—Has had some sleep. The bowels were moved shortly after the exhibition of the first draught, and accordingly the medicine has been discontinued. Pulse eighty, moderately full and soft; some heat of skin; slight tenderness of the abdomen; expression of the countenance calm and natural.

The subsequent progress of the case was so regular and favourable as to require little surgical interference. By August 20th, the wound was perfectly cicatrised; and on the 5th September he left the hospital, having previously been furnished with a truss.

His diarrhœa returned on the day, or second day, after the operation, which Mr. Tyrrell considered as a very favourable circumstance.

The anxious and exhausted state of the patient before the operation was unusually great, so as to preclude entirely any means short of operation: this might have arisen from his previous suffering from diarrhœa.

